# Role of imaging in management of hepatocellular carcinoma: surveillance, diagnosis, and treatment response

**DOI:** 10.20517/2394-5079.2020.42

**Published:** 2020-08-27

**Authors:** Azeez Osho, Nicole E. Rich, Amit G. Singal

**Affiliations:** 1 Department of Internal Medicine, UT Southwestern Medical Center, Dallas, TX 75390-8887, USA.; 2 Division of Digestive and Liver Diseases, UT Southwestern Medical Center, Dallas, TX 75390-8887, USA.

**Keywords:** Liver cancer, ultrasound, screening, computed tomography, magnetic resonance imaging, contrast-enhanced ultrasound, Liver Imaging Reporting and Data System

## Abstract

Imaging plays a notable role in hepatocellular carcinoma (HCC) surveillance, diagnosis, and treatment response assessment. Whereas HCC surveillance among at-risk patients, including those with cirrhosis, has traditionally been ultrasound-based, there are increasing data showing that this strategy is operator-dependent and has insufficient sensitivity when used alone. Several novel blood-based and imaging modalities are currently being evaluated to increase sensitivity for early HCC detection. Multi-phase computed tomography (CT) or contrast-enhanced magnetic resonance imaging (MRI) should be performed in patients with positive surveillance tests to confirm a diagnosis of HCC and perform cancer staging, as needed. HCC is a unique cancer in that most cases can be diagnosed radiographically without histological confirmation when demonstrating characteristic features such as arterial phase hyperenhancement and delayed phase washout. The Liver Imaging Reporting and Data System offers a standardized nomenclature for reporting CT or MRI liver findings among at-risk patients. Finally, cross-sectional imaging plays a critical role for assessing response to any HCC therapy as well as monitoring for HCC recurrence in those who achieve complete response.

## INTRODUCTION

Hepatocellular carcinoma (HCC) is the most common primary liver cancer and the fourth leading cause of cancer-related death worldwide^[1]^. Common risk factors for the development of HCC include alcohol use, chronic hepatitis B (HBV) or hepatitis C infection, and nonalcoholic fatty liver disease (NAFLD). Prognosis for HCC depends on tumor stage at diagnosis; curative treatment options for early stage tumors provide 5-year survival exceeding 70%, whereas late stage HCC is only amenable to palliative therapies with a median survival of 2-3 years. Imaging plays a central role in the management of patients with HCC, including surveillance, diagnosis, and assessing treatment response. The aim of this review is to discuss best practices for imaging along the care spectrum of HCC.

## ROLE OF IMAGING FOR HCC SURVEILLANCE

Given the strong association between early detection and improved survival, the American Association for the Study of Liver Diseases (AASLD) and European Association for the Study of Liver (EASL) recommend HCC surveillance in at-risk patients, including subgroups with chronic HBV and those with cirrhosis from any etiology^[2,3]^. HCC surveillance is supported by a large randomized controlled trial (RCT) in patients with HBV that showed a 37% reduction in mortality^[4]^. Although there is no similar RCT among patients with cirrhosis, several cohort studies have highlighted an association between surveillance and improved early detection, curative treatment receipt, and overall survival^[5]^.

### Ultrasound for HCC surveillance

The preferred imaging modality for HCC surveillance across all major professional liver organizations worldwide has been, and remains, abdominal ultrasound^[2,3,6,7]^. Ultrasound has many advantages including being readily available, inexpensive, and non-invasive with a favorable safety profile. A systematic review of test modalities for HCC surveillance found that ultrasound has a high sensitivity of 94% to detect HCC at any stage; however, its sensitivity to detect early stage HCC is significantly lower at only 63%^[8]^. Furthermore, the wide variation in ultrasound sensitivity between studies highlights the operator-dependent nature of the examination. High ultrasound quality relies heavily on the experience of the individual performing the ultrasound examination as well as the radiologist interpreting the examination^[9,10]^. These challenges have been observed in breast cancer screening, with ultrasound being more useful than mammography in women with dense breast tissue, but one of its limitations being variable quality based on inherent operator dependence^[11]^. Standardization of examination technique and establishment of minimum reporting requirements, as has been done for breast ultrasonography^[12]^, can improve the quality of ultrasound-based screenings^[13]^. For HCC, regional differences have been observed in ultrasound sensitivity and align with differences in technique^[8]^. In the U.S., ultrasound is typically performed by technicians with select frozen images interpreted by a radiologist at a later time, whereas physicians in other regions of the world often perform and interpret ultrasound in real time^[14]^. Recent data have also highlighted the impact of patient characteristics on ultrasound effectiveness. In a retrospective cohort study of 941 patients undergoing surveillance ultrasound, 191 (20.3%) were deemed to be of inadequate quality for exclusion of HCC lesions^[15]^. In multivariable analysis, inadequate ultrasound quality was associated with obesity and alcohol- or nonalcoholic steatohepatitis (NASH)-related cirrhosis, suggesting that inadequate ultrasound quality and poor sensitivity may be more common as the prevalence of obesity and NASH continue to rise globally^[16,17]^. Since this study, the Liver Imaging Reporting and Data System (LI-RADS) has proposed that ultrasound assessment and reporting include an ultrasound visualization score, including score A (no or minimal limitation), score B (moderate limitations that may obscure small masses), and score C (severe limitations that significantly lower sensitivity for focal liver lesions). The visualization score is based on liver heterogeneity, beam attenuation or shadowing, proportion of liver visualized, and proportion of diaphragm visualized. Routine reporting of visualization is an important step that helps clinicians interpret ultrasound results; however, further data are needed to verify that poor visualization is in fact associated with lower HCC detection as well as determining the optimal surveillance strategies in patients with limited visualization. A recent pilot study suggests that repeat ultrasound in patients with limited visualization (scores B or C) could have sufficient visualization (score A) in approximately half of cases; however, validation of these results are needed in larger cohorts^[18]^.

Various surveillance intervals have been proposed^[19]^. The AASLD and EASL recommend semi-annual surveillance, which appears reasonable on the basis of the median doubling time of HCC tumors^[20]^. A retrospective multicenter study among 649 HCC patients from Italy found patients detected by semi-annual surveillance had smaller tumor burden and improved survival compared to patients submitted to annual surveillance (40.3 months *vs*. 30 months, respectively, *P* = 0.03)^[21]^. An RCT evaluated if shorter intervals would further improve early detection and survival but found that a 6-month surveillance interval provided similar early HCC detection compared to a 3-month surveillance interval (79% *vs*. 70%, *P* = 0.30)^[22]^.

### Role of biomarkers for HCC surveillance

Professional societies offer differing guidance regarding the additional value of serum biomarkers over ultrasound alone for HCC surveillance. The best studied biomarker for HCC surveillance is alpha fetoprotein (AFP), which has been validated in all five phases of biomarker development^[23]^. The AASLD and Asian Pacific Association for the Study of the Liver (APASL) both recommend ultrasound with or without AFP^[2]^, whereas the EASL recommends ultrasound alone, citing the poor performance characteristics of AFP^[2,3,6,7]^. The AASLD and APASL guidelines cite the improvement in sensitivity when adding AFP to ultrasound in clinical practice^[8]^. Various AFP cutoffs have been proposed, with the AASLD and APASL recommending AFP cutoffs of 20 and 200 ng/mL, respectively^[2]^. Notably, AFP levels can be increased in patients with active hepatitis, and thus, AFP is less accurate in patients with active HCV infection, whereas AFP has higher sensitivity in other subgroups (e.g., patients with cirrhosis and HIV infection^[24,25]^. In contrast, AFP has better sensitivity in individuals with HBV and HCV, who are either receiving or have completed antiviral treatments, and therefore, lower threshold values can be established in using AFP for surveillance in this patient population^[26,27]^.

Although AFP alone has poor sensitivity for early stage HCC and poor specificity in patients with viral hepatitis, several studies have suggested a potential benefit of using AFP as an adjunct surveillance test with ultrasound. A meta-analysis of cohort studies on this topic demonstrated that the combination of ultrasound and AFP had a significantly higher sensitivity for early stage HCC compared to ultrasound alone (63% *vs*. 45%, respectively^[8]^. Although this was associated with a drop in specificity (92% for ultrasound alone *vs*. 84% for ultrasound plus AFP), this was not felt to be clinically significant and the diagnostic odds ratio of the combination remained higher using the two tests together 7(95%CI: 3-15) *vs*. 8(95%CI: 3-23, respectively). A study by Atiq and colleagues quantified physical harms related to ultrasound with or without AFP, as not all false positive lesions may prompt diagnostic evaluation^[28]^. They found 1 in 4 patients with cirrhosis experience physical harms for false positive or indeterminate surveillance tests, which are more often related to ultrasound than AFP monitoring - in part related to some patients having diagnostic evaluation for indeterminate ultrasound results and providers not ordering diagnostic evaluation in many patients with false positive AFP levels.

Other biomarkers, such as lens culinaris agglutinin-reactive AFP (AFP-L3) and des-gamma-carboxyprothrombin (DCP), have been evaluated in phase 2 (case-control) biomarker studies but appear to have insufficient performance if used alone^[23]^. Therefore, there has been increasing interest in biomarker panels, such as GALAD, which combines AFP, AFP-L3, and DCP with patient age and gender, and has been shown to have promising performance in large case-control studies^[29,30]^. In a large multi-national case-control study with 6,834 patients (2,430 HCC and 4,404 controls), GALAD demonstrated a sensitivity of 60%-80% for early HCC detection. A recent study by Yang *et al*.^[31]^ compared GALAD to ultrasound for HCC detection and found GALAD to be superior to ultrasound, with an area under the curve (AUC) of 0.95 (95%CI: 0.93-0.97) *vs*. 0.82 (95%CI: 0.77-0.87), respectively. When the GALAD model was combined with ultrasound, the GALADUS score had significantly better performance compared to ultrasound alone with a sensitivity of 95%, specificity of 91% and an AUC of 0.98 (95%CI: 0.96-0.99). In addition, there has also been increased interest in methylated DNA markers and circulating tumor cells (CTCs) for early detection of HCC^[32,33]^. However, these biomarkers still require evaluation in large phase II and phase III studies before adoption in clinical practice^[34]^.

### CT/MRI for HCC surveillance

Given the limitations of ultrasound-based surveillance, there has been increasing interest in alternative imaging modalities, such as computed tomography (CT) or magnetic resonance imaging (MRI), but neither is recommended by current practice guidelines^[2,3,6,7]^. Although both CT and MRI have been shown to be superior in sensitivity and specificity for HCC diagnosis and staging compared to ultrasound (discussed below), there are limited data evaluating these tests in a surveillance manner. A small randomized trial comparing semi-annual ultrasound to annual multiphase CT found that ultrasound was similar in sensitivity but less costly than CT^[35]^. Further, CT is associated with screening harms including radiation exposure and potential contrast injury^[36,37]^. Therefore, there has been increasing interest in MRI surveillance, which obviates some of these concerns. A prospective cohort study from South Korea (PRIUS study) comparing ultrasound and MRI surveillance in patients with cirrhosis found that MRI had significantly higher sensitivity for early stage HCC (86% *vs*. 27.9%) as well as higher specificity (97% *vs*. 94.4%)^[38]^. The authors of this study subsequently performed a cost-effectiveness analysis, suggesting that MRI may be cost-effective; however, these data still require validation in non-HBV Western populations^[39]^. Furthermore, there would be concerns about radiologic capacity and patient acceptability if an MRI-based strategy were adopted in larger populations. There have been increasing data on alternative MRI strategies, including abbreviated MRI and non-contrast MRI. Abbreviated MRI protocols use selected sequences from a full diagnostic protocol and can shorten the examination from ~45 min to ~15 min, which may address some concerns about radiologic capacity and improve cost-effectiveness^[40]^. Abbreviated MRI protocols have been studied for HCC diagnosis and characterization of lesions^[40,41]^, but no trials or studies have been done specifically for surveillance. There is an ongoing clinical trial at Seoul National University Hospital comparing annual abbreviated MRI to ultrasound for early HCC detection (NCT03731923). Two recent studies have also evaluated non-contrast MRI as a possible surveillance strategy. A *post-hoc* analysis of the PRIUS study suggested that non-contrast MRI is superior to ultrasound for HCC detection, with per-lesion and per-examination sensitivity of 77.1% and 79.1% for non-enhanced MRI compared to just 25.0% and 27.9% for ultrasound, respectively^[42]^. Specificity of non-contrast MRI was also higher than that of ultrasound 97.9% *vs*. 94.5%, *P* < 0.001]. In addition, the estimated scan time was < 6 min with a total room occupancy time of only 25-35 min. Two ongoing prospective trials, MIRACLE-HCC (NCT02514434) and MAGNUS-HCC (NCT02551250), are comparing non-contrast MRI and ultrasound for surveillance of HCC^[43,44]^.

Most analyses for HCC surveillance have tried to implement a “one-size-fits-all” strategy for all at-risk patients, despite known variation in HCC risk between patients with cirrhosis. For example, a validated tissue-based signature has been shown to accurately risk stratify patients with cirrhosis into high, intermediate, and low risk of HCC, with annual HCC incidences of 5.8, 2.2, and 1.5%, respectively^[45]^. Similarly, other risk stratification markers can accurately distinguish patients with high risk and low risk of developing HCC^[46]^. Accurate risk stratification could allow more intensive and costly surveillance strategies to be applied to those at highest risk, while using lower intensity and inexpensive surveillance strategies in lower risk patients. A modeling study suggested that a risk-stratified approach was cost-effective compared to ultrasound and AFP in all patients^[47]^. Currently, the Japan Society of Hepatology (JSH) is the only professional society that recommends a differential HCC surveillance strategy by individual patient risk, i.e., ultrasound and serum biomarkers for most patients, with multiphase CT or MRI considered in the highest risk patients^[7]^.

Unfortunately, a systematic review found that less than 20% of patients with cirrhosis in the U.S. undergo HCC surveillance, with even lower rates of guideline-concordant semi-annual surveillance^[48,49]^. Patients and providers have reported potential barriers to surveillance including knowledge deficits, time constraints, and financial costs of tests that need to be addressed to increase surveillance utilization^[50,51]^. Studies have demonstrated promise for inreach efforts such as electronic medical record reminders or outreach strategies including mailed invitations to complete ultrasound surveillance^[52-54]^.

While awaiting ongoing trial data for both novel biomarkers and cross-sectional imaging techniques, ultrasound with or without AFP remains the gold standard surveillance strategy.

## ROLE OF IMAGING IN HCC DIAGNOSIS

**Figure 1.**
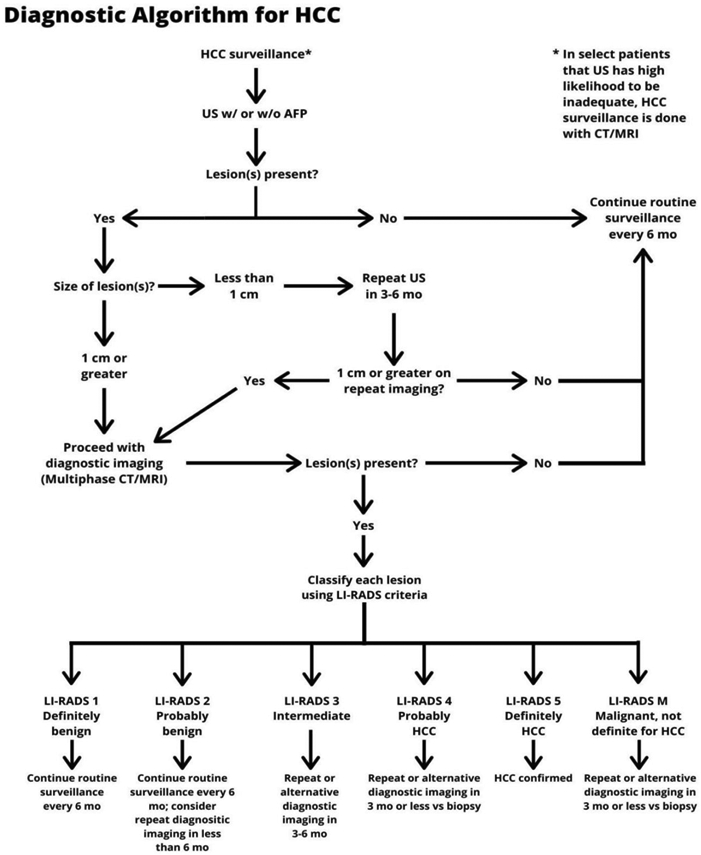
Diagnostic algorithm for HCC. *In select patients in whom US has high likelihood to be inadequate, HCC surveillance may be performed using contrast-enhanced CT or MRI. HCC: hepatocellular carcinoma; CT: computed tomography; MRI: magnetic resonance imaging; US: ultrasound

For surveillance to be effective, recall procedures must be followed for patients with abnormal surveillance tests^[55]^. In patients with an ultrasound nodule < 1 cm in maximum diameter, the risk of HCC is low and professional society guidelines recommend repeat short-interval ultrasound in ~3 months. If the lesion is stable in size, it can be followed on ultrasound; however, diagnostic evaluation with cross-sectional imaging (i.e., contrast-enhanced MRI or 4-phase CT) is recommended once a lesion is ≥ 1 cm in size^[2]^ [Figure 1].

HCC is unique compared to other cancers, in that the diagnosis can often be made radiographically without histological confirmation. Historically, HCC diagnosis has been made on the basis of the presence of “arterial enhancement and delayed washout” i.e., hypervascularity during the arterial phase and hypointensity on the portal venous or delayed phases of imaging. This classic appearance is related to the liver’s dual blood supply, where the background liver receives most of its blood supply from the portal vein and HCC lesions obtain most of their blood supply from hepatic artery branches. In the setting of cirrhosis, this appearance was demonstrated to have a specificity of 95% for the diagnosis of HCC^[56,57]^.

### LI-RADS criteria

More recently, the American Association for Cancer Research (AACR) and AASLD have adopted the LI-RADS criteria for HCC diagnosis and classification, and have chosen specific populations for which these criteria should be applied, namely patients with cirrhosis and chronic hepatitis B infection^[57]^. The LI-RADS criteria do not apply to pediatric patients or patients with cirrhosis secondary to vascular disorders (e.g., Budd-Chiari syndrome, sinusoidal obstruction syndrome)^[58]^. The LI-RADS criteria include a combination of major and minor imaging criteria, and classifies lesions on a scale ranging from LR-1 (definitely benign) to LR-5 (definitely HCC) or LR-M (malignant but not definite for HCC) [Table 1]. Major LI-RADS criteria include arterial phase hyperenhancement (APHE), delayed washout, enhancing capsule, and threshold growth. Patients with LR-1 and LR-2 lesions are definitely and likely benign, respectively, so most of these patients can return to ultrasound-based surveillance. Patients with LR-3 and LR-4 lesions have an intermediate risk of HCC, so these patients can be considered for continued observation versus biopsy after multidisciplinary discussion. A recent systematic review found 38 and 74% of LR-3 and LR-4 lesions were HCC, respectively, highlighting that these lesions should not be ignored and must be followed clinically^[59]^. In this systematic review, LR-5 lesions had a positive predictive value of 94% for being HCC, and therefore do not warrant biopsy for histological confirmation prior to treatment. The LR-M classification is reserved for lesions that are suspicious for malignancy but have features that are not definite for HCC, e.g., peripheral enhancement, and can be seen in other malignancies such as intrahepatic cholangiocarcinoma. Therefore, biopsy is typically recommended in these cases to make a definitive diagnosis. It should also be noted that the LI-RADS criteria do not apply to patients without cirrhosis and/or chronic HBV infection, as the positive predictive value of the aforementioned classic imaging findings is substantially lower^[60]^. Therefore, these patients should typically undergo biopsy for histological confirmation prior to treatment^[2]^.

### CT/MRI for HCC diagnosis

Several studies have compared the accuracy of CT and MRI for the diagnosis of HCC. In a meta-analysis by Roberts *et al*.^[61]^ contrast-enhanced CT was compared to both extracellular contrast-enhanced MRI and Eovist MRI for HCC diagnosis. Compared to CT, MRI had a significantly higher sensitivity (82% *vs*. 66%) with similar specificity (92% *vs*. 91%). In addition, MRI was more sensitive for diagnosis of HCC in lesions < 1 cm compared to CT (69% *vs*. 49%), although specificity was lower (46% *vs*. 69%, respectively). Conversely, MRI was associated with higher cost, greater technical complexity (including longer scan time), and less consistent imaging quality (e.g., difficulty with breath holding, difficulty holding still, large volume ascites). Thus, although MRI was noted to have marginally higher sensitivity compared to CT in this meta-analysis, one imaging modality could not be definitively recommended over the other, and the choice of modality should be individualized considering both the risks of either imaging test and the patient’s clinical status^[61]^.

Although contrast-enhanced MRI and 4-phase CT are the primary modalities used for HCC diagnosis in the Western world, APASL guidelines recommend the use of hepatobiliary agents (e.g., gadoxetic acid or Eovist), which can provide information on hepatocellular function in addition to blood flow. This recommendation is largely based on data suggesting increased sensitivity to detect HCC lesions compared to dynamic CT and MRI. A 2015 meta-analysis compared the diagnostic performance of dynamic CT, MRI using conventional extracellular contrast agents, and MRI using hepatobiliary contrast agents. Overall, on a per-lesion basis, MRI was more sensitive than CT for HCC diagnosis (80% *vs*. 68%, *P* = 0.02). In subgroup analyses, the per-lesion sensitivity of gadoxetic acid-enhanced MRI was significantly higher compared to MRI using other contrast agents (87% *vs*. 74%, *P* = 0.03)^[62]^. However, gadoxetic acid-based MRI has some limitations in the diagnosis of HCC. While HCC are usually hypointense on the hepatobiliary phase (due to lack of contrast uptake by the tumor compared to the background liver showing peak enhancement at this time), up to 20% of HCCs will have uptake of the contrast and instead appear hyperintense^[63]^. Additionally, the classic feature of “pseudocapsule” may not be apparent due to lack of a delayed phase, leading to misdiagnosis^[6]^. Furthermore, in patients with more advanced cirrhosis, decreased contrast uptake in the background liver may lead to lower sensitivity for HCC detection^[64]^.

### PET for HCC diagnosis

The use of positron-emission tomography (PET) has been evaluated for diagnosis of HCC but has not produced favorable results for detection of primary tumors. The most widely used radiotracer is ^18^F-fluorodeoxy-6-glucose phosphate (FDG), which has added utility in assessing metabolic cellular function, but ^18^F-FDG uptake in PET/CT for primary HCC has only been seen in 40% of cases^[65]^. This is due to high ^18^F-FDG uptake by both normal hepatocytes and malignant neoplastic cells associated with HCC, resulting in difficulty in identifying HCC lesions^[66,67]^. However, PET may be beneficial for diagnosis of extrahepatic or metastatic HCC. One of the most common sites for extrahepatic HCC metastasis is the retroperitoneal lymph nodes, and the sensitivity of FDG PET/CT to detect lymph node metastasis is greater than in other areas of the body^[68]^. Other forms of PET imaging that have been studied for HCC diagnosis include PET MRI and immuno-PET/CT. PET MRI has the benefit of improved soft tissue contrast and a lack of ionizing radiation. However, its availability is limited and requires a technologist experienced in both nuclear medicine and MRI for accurate interpretation^[69]^. Immuno-PET/CT uses ^89^Zr-tagged monoclonal antibodies to target glypican-3, a cell surface protein that is highly expressed in HCC, and has shown improvement in differentiating primary HCC cells from normal hepatocytes and identifying small HCC lesions compared to PET alone^[65]^. However, studies evaluating immuno-PET have been limited to animal models, and further studies are needed before its routine use in clinical practice^[70]^.

### Contrast-enhanced ultrasound for HCC diagnosis

There has also been increasing interest in the role of contrast-enhanced ultrasound (CEUS) for HCC diagnosis. This imaging modality uses the intravenous administration of microbubble contrast agents to evaluate the hyperenhancement of a liver nodule in “real-time” These contrast agents have a short half-life of only a few minutes and are eliminated through respiration, eliminating concerns for potential renal toxicity seen with most contrast agents used for CT and MRI^[71]^. The LI-RADS criteria have been modified for using CEUS for characterization of liver nodules, similar to the LI-RADS criteria for CT/MRI^[72]^. A meta-analysis showed that the pooled sensitivity and specificity of CEUS to detect HCC was 85 and 91%, respectively; however, the authors noted the findings were limited by publication bias^[73]^. There are several notable limitations of CEUS that are similar to conventional ultrasound in HCC diagnosis. First, ultrasound is operator-dependent, which may lead to inconsistencies in diagnosis outside of expert centers^[74]^. Second, CEUS can also be limited by patient-level factors, including large body habitus, overlying bowel gas, poor acoustic windows, and movement artifact^[72,74]^. A limitation of CEUS in HCC diagnosis that differs from conventional ultrasound involves the nuances of contrast administration to properly characterize suspicious lesions. Multiple injections of contrast may be needed to properly classify lesions, thereby limiting its role for staging, and the administration of contrast must be done in a medically controlled setting to ensure safety^[74]^. Lastly, CEUS has lower detection rate for washout than CT/MRI^[75]^, and its ability to distinguish HCC from intrahepatic cholangiocarcinoma (ICC) has been controversial^[76,77]^. However, some studies have suggested that dynamic, timed administration of contrast can be used in CEUS to help distinguish the two malignancies, as the rapid loss of signal intensity in the early portal phase is more characteristic of ICC than HCC^[78]^. Additional criteria have been proposed to distinguish ICC and HCC using CEUS with reported improved performance but require further validation^[79]^. Based on current practice guidelines, CEUS is reserved as a second-line diagnostic imaging modality when multiphase CT or MRI are indeterminate in HCC diagnosis, although data continue to evolve regarding its potential role^[7]^.

## ROLE OF IMAGING FOR POST-TREATMENT RESPONSE AND SURVEILLANCE

Patients with early stage HCC are typically eligible for curative therapies including local ablation, surgical resection, or liver transplantation. Although resection and local ablation are considered curative, they are associated with a high risk of recurrence, approaching up to 70% at 5 years^[80]^. Therefore, close observation is critical, with most centers performing CT or MRI every 3 months for the first 1-2 years and then semi-annual surveillance with CT or MRI thereafter. Some centers return to ultrasound-based surveillance after a period of 4-5 years, although there is substantial center-to-center variation. Liu and colleagues used clinical and tumor features to risk stratify patients into 3 categories (low, intermediate, and high risk of recurrence) following surgical resection to determine the optimal time interval for post-hepatectomy surveillance imaging^[81]^. They calculated recurrence detection rates between consecutive CT for each surveillance schedule for each risk group, and found surveillance schedules could be tailored on the basis of risk; for example, low-risk patients could undergo surveillance CT every four months for the first two years and yearly over the next three years without compromising surveillance benefits while reducing examination costs and radiation burden.

### Surveillance after liver transplantation

Liver transplantation has the advantage of curing not only HCC but also the underlying cirrhosis, and is thus associated with significantly lower recurrence rates (~10% at 5 years) when restricted to patients within the Milan criteria on imaging (one lesion < 5 cm or 2-3 lesions each < 3 cm, without vascular invasion or extrahepatic spread)^[82]^. More recently, liver transplant criteria have been expanded to include patients who are “downstaged” i.e., patients with larger tumor burden who are treated with locoregional therapy (LRT) and brought to within Milan criteria. Radiographic response is used as a prognostic biomarker and serves as a surrogate for tumor biology, with those who exhibit response likely having favorable tumor biology. Several single and multicenter studies have shown similar survival and rates of post-transplant recurrence among extended-criteria patients who were successfully downstaged with LRT compared to those who initially presented within Milan criteria^[83,84]^. In the largest multicenter study to date including patients with HCC from 10 of 11 UNOS regions that underwent liver transplantation, Kardashian *et al*.^[85]^ found 5-year overall survival to be acceptable in patients downstaged to within Milan criteria compared to those initially within Milan criteria (64% *vs*. 71%). In addition, the authors noted that AFP response to LRT provided a useful adjunct to radiographic response in assessing likelihood of successful downstaging^[85]^. Post-transplant, HCC surveillance is evolving from a one-size-fits-all strategy to a tailored one based on an individual’s risk of recurrence. The Risk Estimation of Tumor Recurrence After Transplant (RETREAT) score developed and validated in a multicenter study by Mehta and colleagues includes 3 variables - AFP at time of transplantation, presence of microvascular invasion, and the largest viable tumor diameter (cm) plus the number of viable tumors on explant pathology^[86]^. Patients are assigned a risk score of 0-8 based on the presence or absence of these features. The RETREAT score accounts for the effect of pre-transplant LRT (as only viable tumor on explant is counted) and stratifies 5-year HCC recurrence risk - noted to be < 3% in patients with a score of 0 to > 75% in patients with a score ≥ 5. Post-operative imaging surveillance intervals can then be tailored on the basis of an individual patient’s RETREAT score. For instance, a patient with a RETREAT score of five might undergo surveillance with CT chest and abdomen every three to four months for the first two years followed by every six months through year five, while patients with a RETREAT score of zero might not require post-transplant imaging surveillance at all.

### Surveillance after locoregional therapies

LRTs, including ablation, transarterial chemoembolization, transarterial radioembolization, and radiation therapy, are a standard treatment for patients with early to intermediate stage HCC who are not candidates for surgical resection or liver transplantation. Furthermore, as previously mentioned, LRT also has a role in downstaging and “bridging” patients to surgical treatments including transplantation. Response to LRT is typically assessed radiographically using CT or MRI, with serum biomarkers used as adjuncts. One of these such systems is known as the Response Evaluation Criteria in Solid Tumors (RECIST), which uses tumor size and characteristics, involvement of lymph nodes, maximum number of target lesions, and disease progression to qualify treatment response for malignancy^[87]^. The use of RECIST has several limitations for HCC response assessment, as it does not consider tumor necrosis nor decrease in tumor size in HCC treated with LRT, and antitumor activity may be poorly correlated. To overcome these limitations, EASL recommended measuring change in area of tumor enhancement to assess treatment response, and in 2008, the AASLD proposed modified RECIST (mRECIST) criteria to include change in lesion size, lesion characteristics, and viable portions of the lesions determined by arterial phase enhancement to determine the response to treatment^[88,89]^. Both RECIST and mRECIST criteria classify treatment response for HCC lesions as complete, partial, stable disease, progressive disease, or development of new lesion(s). Overall response by both the EASL and mRECIST criteria have been associated with survival and are thus preferable to RECIST for HCC response assessment^[90]^. Still, mRECIST has some notable limitations. First, the response assessment requires radiologic expertise as ascertainment of viable tumor may not be straightforward. Second, patients with underlying renal disease or impairment may be unable to tolerate a contrast-enhanced examination and, therefore, mRECIST evaluation^[91]^. In addition, timing of contrast administration and imaging acquisition must be precise to prevent misinterpretation. LI-RADS has also proposed a nomenclature for reporting response, categorizing patients as having residual disease (LR-TR viable), having complete response with no viable tumor (LR-TR non-viable), or situations in which it is unclear if there is viable tumor remaining (LR-TR equivocal). There are few, if any, data comparing LI-RADS response assessment to other response assessments such as mRECIST.

### Surveillance after systemic therapy

Systemic therapy is the mainstay of treatment for patients with advanced HCC, and valid radiologic response criteria are critical for the assessment of treatment response in clinical trials. Sorafenib, an oral multikinase inhibitor, was the first chemotherapy agent approved for first-line treatment of HCC in the U.S. on the basis of data from the multicenter, randomized, double-blind placebo-controlled Sorafenib Hepatocellular Carcinoma Assessment Randomized Protocol (SHARP) trial^[92,93]^. Since 2017, several additional tyrosine kinase inhibitors (TKIs) for HCC have been approved for first- and second-line indications, including lenvatinib, regorafenib and cabozantinib^[94-96]^. A study by Edeline *et al*.^[97]^ compared RECIST to mRECIST in patients receiving sorafenib for treatment of advanced HCC and found mRECIST objective response correlated with an increased overall survival. Similarly, Kudo *et al*.^[98]^ demonstrated that objective response per mRECIST was associated with improved survival in a post-hoc analysis of the REFLECT Trial including patients treated with sorafenib or lenvatinib. Median overall survival for patients with an objective response was 22.4 months, compared to 11.4 months for non-responders^[98]^. Most recently, Llovet and colleagues evaluated 21 clinical trials in HCC and found a moderate correlation between progression-free survival or time to progression and overall survival; however, a hazard ratio of ≤ 0.6 appeared to be a potential surrogate for improved survival^[99]^. Overall, these data highlight the importance of monitoring for both response and progression.

With the introduction of checkpoint inhibitors (e.g., nivolumab, pembrolizumab, atezolizumab/bevacizumab), durable response rates in approximately 15%-30% of patients have been observed^[100]^. In an exploratory analysis of data from Checkmate 040, patients with durable objective responses appeared to have prolonged survival compared to those with stable disease or progressive disease^[101]^. However, it is possible some patients treated with immunotherapy may display “pseudoprogression”, a distinct radiologic pattern in which deep and durable responses occur after initial progression^[102,103]^. Although this is well described for other tumor types, e.g., melanoma, it is currently unclear how commonly this occurs in patients with HCC. This phenomenon has resulted in the development of specific imaging response assessment guidelines (irRECIST and iRECIST) for this population, in which radiographic progression must be confirmed with repeat imaging 4-8 weeks after the first response assessment^[104,105]^. These various response assessment classifications are being used in ongoing HCC clinical trials and there has yet to emerge a standard across all trials^[106]^. Despite this potential uncertainty regarding optimal ways to assess response, monitoring for treatment response or disease progression can identify patients who are benefiting from therapy and those who may benefit from transition to an alternative treatment. Our institutional practice is to monitor patients with cross-sectional imaging every 2 months while on systemic therapy.

## FUTURE DIRECTIONS FOR IMAGING IN HCC

Radiomics, the automated high-throughput extraction and analysis of quantitative and phenotypic features from radiographic images^[107]^, has emerged as a non-invasive tool for diagnosis and prognostication in several cancers, including HCC^[108]^. Qualitative and quantitative radiomics features may predict HCC recurrence and treatment response^[109]^, and are promising as novel biomarkers that may be complementary to existing serum biomarkers for HCC surveillance and treatment response assessment. However, lack of reproducibility is a major challenge and further validation studies are needed prior to the adoption of radiomics in routine clinical practice.

## SUMMARY

Imaging has played a significant role in the advancements of surveillance, diagnosis, and treatment of HCC. Across all professional societies, ultrasound is the most recognized imaging modality for HCC screening among at-risk patients. CT and MRI are currently not recommended for surveillance given similar sensitivities as ultrasound and cost-effectiveness, but recent trials are studying abbreviated MRI protocols for surveillance. Non-invasive diagnosis of HCC relies heavily on CT and MRI with application of the LI-RADS in classifying suspicious lesions for HCC. PET imaging is best utilized to identify extrahepatic metastases but has poor performance for diagnosis of primary HCC. CEUS has also been studied for its role in HCC diagnosis and is currently accepted as a second line imaging modality in most professional societies. Imaging with CT and MRI has also been shown to be effective in monitoring treatment response, with most centers using RECIST or mRECIST for trial analysis.

## Figures and Tables

**Table 1.** LI-RADS classification for liver lesions

Non-rim arterial phase enhancement		Absent		Present		
Observed size of lesion (mm)		< 20	≥ 20	< 10	10-19	≥ 20
Presence of additional major features	None	LR-3	LR-3	LR-3	LR-3	LR-4
Enhancing “capsule”	One	LR-3	LR-4	LR-4	LR-4/LR-5*	LR-5
Nonperipheral washout	≥ Two	LR-4	LR-4	LR-4	LR-5	LR-5
Threshold growth						

* If a lesion is classified in this category and has enhancing capsule, it is categorized as LR-4. However, if a lesion is classified in this category and has either nonperipheral washout OR threshold growth, it is classified as LR-5. LI-RADS: Liver Imaging Reporting and Data System
